# Mutation screen and association studies in the Diacylglycerol O-acyltransferase homolog 2 gene (*DGAT2*), a positional candidate gene for early onset obesity on chromosome 11q13

**DOI:** 10.1186/1471-2156-8-17

**Published:** 2007-05-03

**Authors:** Susann Friedel, Kathrin Reichwald, André Scherag, Harald Brumm, Anne-Kathrin Wermter, Hans-Rudolf Fries, Kerstin Koberwitz, Martin Wabitsch, Thomas Meitinger, Matthias Platzer, Heike Biebermann, Anke Hinney, Johannes Hebebrand

**Affiliations:** 1 Department of Child and Adolescent Psychiatry, University of Duisburg-Essen, Virchowstr. 174, 45147 Essen, Germany; 2 Institute of Medical Biometry and Epidemiology, Philipps-University, Bunsenstr. 3, 35037 Marburg, Germany; 3 Department of Pediatric Endocrinology, Charite Children's Hospital, Humboldt University, Augustenburger Platz 1, 13353 Berlin, Germany; 4 Clinical Research Group, Department of Child and Adolescent Psychiatry, Philipps-University, Schuetzenstr. 49, 35037 Marburg, Germany; 5 Chair of Animal Breeding, Technical University Munich, Hochfeldweg 1; 85350 Freising-Weihenstephan, Germany; 6 GSF National Research Center for Environment and Health, Ingolstaedter Landstr. 1, 85764 Neuherberg, Germany; 7 Department of Pediatrics and Adolescent Medicine, University of Ulm, Eythstraße 24, 89081 Ulm, Germany; 8 Genome Analysis, Leibniz Institute for Age Research – Fritz Lipmann Institute, Beutenbergstrasse 11, 07745 Jena, Germany

## Abstract

**Background:**

*DGAT2 *is a promising candidate gene for obesity because of its function as a key enzyme in fat metabolism and because of its localization on chromosome 11q13, a linkage region for extreme early onset obesity detected in our sample.

We performed a mutation screen in 93 extremely obese children and adolescents and 94 healthy underweight controls. Association studies were performed in samples of up to 361 extremely obese children and adolescents and 445 healthy underweight and normal weight controls. Additionally, we tested for linkage and performed family based association studies at four common variants in the 165 families of our initial genome scan.

**Results:**

The mutation screen revealed 15 DNA variants, four of which were coding non-synonymous exchanges: p.Val82Ala, p.Arg297Gln, p.Gly318Ser and p.Leu385Val. Ten variants were synonymous: c.-9447A > G, c.-584C > G, c.-140C > T, c.-30C > T, IVS2-3C > G, c.812A > G, c.920T > C, IVS7+23C > T, IVS7+73C > T and *22C > T. Additionally, the small biallelic trinucleotide repeat rs3841596 was identified. None of the case control and family based association studies showed an association of investigated variants or haplotypes in the genomic region of *DGAT2*.

**Conclusion:**

In conclusion, our results do not support the hypothesis of an important role of common genetic variation in *DGAT2 *for the development of obesity in our sample. Anyhow, if there is an influence of genetic variation in *DGAT2 *on body weight regulation, it might either be conferred by the less common variants (MAF < 0.1) or the detected, rare non-synonymous variants.

## Background

Obesity has become a major public health problem in industrialized countries and its prevalence is still increasing worldwide [1]. Estimates from twin studies attribute up to 80% of human body weight variation to genetic factors [2] and positional candidate gene analyses in linkage peak regions identified in genome wide scans for obesity have been suggested as a means to detect obesity associated genes [i.e. [3-7]]. Examples for positional candidate gene association findings pertain to (a) *SLC6A14 *on chromosome (chr.) Xq24 [3] which was confirmed by Durand et al. [4] and (b) *GAD2 *on chr. 10p12 [5] which was confirmed by the same group [6]. In contrast, Swarbrick et al. [7] found no evidence for a relationship between the three *GAD2 *SNPs and obesity in a sample comprising 2,359 individuals.

A genome wide scan for obesity based on 89 German families, comprising extremely obese children and adolescents and both of their parents and at least one obese sib, identified nine regions with maximum likelihood binomial logarithm of the odd (MLB LOD) scores > 0.7; in an independent confirmation sample of 76 obesity families MLB LOD scores of 0.68 and 0.71 were observed for chromosomes 10p11.23 and 11q13, respectively [8].

The hypothesis of a susceptibility gene for obesity and related phenotypes on chromosome 11q13 was additionally supported by independent linkage studies for BMI and obesity related phenotypes [9-12]. Further support was obtained from chromosomal regions homologous to human chromosome 11q13 in rodents in which quantitative trait loci (QTL) for obesity related phenotypes such as leptin level [13] and BMI [14] were identified. Taken together, there is evidence for a candidate gene for obesity in this chromosomal region.

In earlier studies, we investigated different promising candidate genes on chr.11q, but none of them contributed to the linkage peak [15-17]. Diacylglycerol-O-acyltransferase homolog 2 (*DGAT2*), another potential candidate gene, is also located on chr. 11q13. DGAT2 is a key enzyme in fat metabolism [18,19]. It is responsible for the synthesis of triglycerides and catalyzes the reaction that joins diacylglycerol covalently to long chain fatty acyl-CoAs. It was hypothesized that leptin regulates adipocyte size by altering expression patterns of Diacylglycerol O-acyltransferase 1 (DGAT1) and its functional homolog DGAT2 via the CNS to determine the levels of triglyceride synthesis [20]. The deduced 387-amino acid human DGAT2 protein contains at least one transmembrane domain, three potential N-linked glycosylation sites, six potential phosphorylation sites, and a putative glycerol phospholipid domain found in acyltransferases [18]. Although functionally related, *DGAT2 *shares no sequence homology with the members of the *DGAT1 *family. The gene was identified via homology search with fungal DGAT subsequent to the finding that *Dgat1 *knockout mice (*Dgat1*^-/-^) were viable and still able to synthesize triglycerides [18,19,21].

*Dgat2 *knockout mice (*Dgat2*^-/-^) are lipopenic, their total carcass triglyceride content was reduced by 93% [22]. In contrast to *Dgat1*^-/- ^mice, where *Dgat2 *is able to compensate the role of Dgat1 in triglyceride synthesis, *Dgat1 *was unable to compensate for the absence of Dgat2 in *Dgat2*^-/- ^mice. *Dgat2*^-/- ^mice die in the early postnatal period, apparently from abnormalities in energy homeostasis and from impaired permeability barrier function in the skin. The results indicate that Dgat2 is the major enzyme of triglyceride synthesis in mice [22].

Based on both positional as well as on functional arguments, we hypothesized that genetic variations in *DGAT2 *might alter triglyceride synthesizing activity of the protein in humans. Genetic variations leading to a gain of function of DGAT2 may thus be associated with obesity, whereas variations entailing a reduced function could be relevant in underweight.

## Results

### Gene structure

To include all potentially relevant exons of *DGAT2*, its structure was analyzed both *in silico *and experimentally. Visual inspection of ESTs assembled to the *DGAT2 *locus in the UCSC genome browser identified two ESTs (BF979495, BF979677) which seemed to harbour alternative/additional exons. The sequences of both ESTs overlap by 200 bp and form a transcript of 1,238 bp. Alignment of this mRNA to genomic DNA revealed the presence of an alternative first noncoding exon of human *DGAT2*, while exons2–8 are as defined by AB048286 (suppl. table 1). Sequencing of EST BF979677 revealed the presence of an alternative internal exon which is located between exon1 and exon2 as defined in AB048286. Furthermore, by RT-PCR in human adipocyte mRNA, a transcript was identified that comprised 7exons in which exon1 and exons3–8 are as defined by AB048286 while exon 2 is missing. In sum, three alternatively spliced transcripts of the human *DGAT2 *gene were identified. Including the two previously reported mRNAs (AB048286, ENST00000228027) there are at least five different mRNAs transcribed from this locus [see additional file 1].

**Table 1:** Summary of DGAT2 variants detected in the coding region, the predicted promoter region and a 5'non-coding exon: 15 (14 novel) identified and 2 previously described (rs1017713 and rs3060), minor allele frequency among all successfully genotyped individuals and results of the case control association studies with cases (extremely obese children and adolescents) and controls (normal- or underweight healthy individuals)

**variant**		**region**	**Study group^1^**	**minor allele frequency n (%)**		**p-value^2^**
					
				**cases**	**controls**	
g.-9447 A > G		exon 01	2	29 (8.06)	33 (8.82)	0.79
c.-584C > G		promoter	1	0 (0)	1 (0.53)	nd.
c.-140C > T		5'UTR/exon 1	3	2 (0.28)	9 (1.01)	0.13
c.-30C > T		5'UTR/exon 1	1	0 (0)	2 (1.06)	nd.
c.475T > C	p.Val82Ala	exon 2	1	1 (0.54)	0 (0)	nd.
g.IVS1+212T > C	rs1017713	exon 2	2	25 (7.65)	26 (7.06)	0.77
g.IVS2-3C > G		intron 2	1	0 (0)	1 (0.53)	nd.
c.812A > G	p.Thr194Thr	exon 5	1	1 (0.54)	0 (0)	nd.
c.920T > C	p.Ser230Ser	exon 6	1	1 (0.54)	0 (0)	nd.
c.1020G > A	p.Arg297Gln	exon 7	1	0 (0)	2 (1.06)	nd.
c.1492G > A	p.Gly318Ser	exon 7	1	0 (0)	2 (1.06)	nd.
g.IVS7+23C > T		intron 7	1	1 (0.54)	0 (0)	nd.
g.IVS7+73C > T		intron 7	1	2 (1.08)	0 (0)	nd.
g.IVS7+164(TAG)2–3	rs3841596	intron 7	2	24 (6.67)	28 (7.61)	0.67
c.1383C > G	p.Leu385Val	exon 8	1	0 (0)	1 (0.53)	nd.
g.*19T > C	rs3060	3'UTR/exon 8	2	27 (7.50)	27 (7.76)	1
g.*22C > T		3'UTR/exon 8	1	1 (0.54)	0 (0)	nd.

Hardy Weinberg equilibrium was fulfilled (all exact *p *> > 0.20). ^1^for descriptions of study groups see Methods; ^2^Fisher's exact test, two-sided.

### Mutation screen

Screening was performed in the coding region, the predicted promoter region and in the identified non-coding 5' exon. The mutation screen in ten fragments comprised 3,079 bps and revealed 15 (14 novel) DNA variants, four of which are coding non-synonymous exchanges: p.Val82Ala, p.Arg297Gln, p.Gly318Ser and p.Leu385Val whereas ten variants are synonymous c.-9447A > G, c.-584C > G, c.-140C > T, c.-30C > T, IVS2–3C > G, c.812A > G, c.920T > C, IVS7+23C > T, IVS7+73C > T and *22C > T (see also table 1). Additionally, a small known biallelic trinucleotide repeat (IVS7+164(TAG)2–3 = rs3841596) located in intron 7 was identified.

### Case control association studies

Minor allele frequencies (MAF) of the variants were estimated in sample 1. Most of the variants were rare and it was thus decided to genotype only the more frequent variations rs3841596, rs1017713 and rs3060 in sample 2. Variant -140C > T, located 5' to the translation start, was genotyped in sample 3 which includes sample 2 but is larger and therefore has an improved power (see table 1). Given the sample sizes, the study had a statistical power of more than 80% to detect allelic differences between the respective case and control groups of e.g. 0.17 and 0.1 in MAFs. Genotype distribution in all study samples did not differ from Hardy-Weinberg equilibrium. No significant differences in genotype or allele distributions were found in samples 2 and 3, all nominal p-values were >> 0.05 (see table 1).

### Family based association studies

To investigate the contribution of *DGAT2 *polymorphisms to the linkage peak on chromosome 11q13 [8] SNPs -9447A > G and -140C > T, as well as two additional known variants (rs1017713 (IVS1+212T > C) and rs3060 (*19T > C)) were genotyped in the families contributing to the genome scan peak (sample 4). Neither single marker family based association analyses (PDT) in all 165 families nor in the 48 families contributing to the linkage peak on 11q13, revealed significant evidence for allelic associations (all p-values > > 0.05). Consistent with this finding, subsequent haplotype analyses using FAMHAP did not indicate an associated haplotype (best nominal p-value 0.5 with the zhaomax allcombi option).

## Discussion

The linkage scan in 89 families revealed the highest LOD at D11S1313. Subsequent fine-mapping in 76 independent families revealed a combined peak region at position 67.8 – 69.1 Mb (approximately 68.55 - 68.01 cM, UCSC, hg16) between D11S1337 and D11S4095 [8, *unpublished data*]. *DGAT2 *is located at 75 Mb and thus close to this peak region. In light of the small sample size, which leads to considerable stochastic variation in the location estimate of linkage peaks [23] and combined with its important role in fat metabolism *DGAT2 *is a very plausible positional and functional candidate gene for obesity in our sample.

A mutation screen in the coding region of the gene, the predicted promoter sequence and a 5' non-coding exon (altogether 3,079 bp) revealed 15 genetic variants, 14 of which were novel. Twelve of the variants were rare (MAF = 1%) and would thus have a too low statistical power to allow for a comparison in a case control association analysis. Nonetheless, these rare variants might have an impact on the phenotype. Four coding non-synonymous variants were detected: p.Val82Ala occurred once in an extremely obese male, whereas p.Arg297Gln, p.Gly318Ser and p.Leu385Val were detected in underweight controls. [1] The conservative amino acid (aa) exchange p.Val82Ala is located in a predicted transmembrane domain of the DGAT2 protein [18]. This position is situated within an area highly conserved among the selected species with Val82 being unchanged for more than 1 billion years of evolution. While this non-synonymous variant seemingly does not affect the predicted transmembrane domain (aa 73 to aa 95), altered function may be the consequence as already postulated for other genes [24]. Moreover, for the very same aa substitution positioned within a transmembrane domain (TM) an inactivating variant in TM2 of the monocarboxylate transporter 8 [25] as well as an activating variant in TM1 of the lutropin receptor [26] had been described. Therefore although Val82Ala is a conservative exchange it has been shown that a Valin to Alanin substitution is able to materially affect membrane protein functions in both an activating as well as in an inactivating manner. Hence, assuming that a gain of function might well lead to obesity, it is reasonable to consider the Valin to Alanin substitution in DGAT2 as a potential cause for the patient's remarkably increased BMI (see table 2). [2] Arg297Gln is a non conservative amino acid exchange. In contrast to arginine, glutamine has an amide-side group that is able to form hydrogen bonds, which might influence protein structure. However, positioned in a region of little evolutionary conservation characterised by a difference in amino acid sequence length between mammals and plants and a non-conservative amino acid exchange between these kingdoms (basic polar arginine in mammals vs. neutral unpolar methionine in plants) an exchange of the wt arginine vs. also polar but neutral glutamine does not suggest a functional consequence of this substitution. [3] The substitution of glycine to serine at position 318 is also non-conservative. During evolution persisted at this position a neutral unpolar amino acid; therefore an exchange by polar serine may be functionally relevant. However, several amino acids flanking position 318 show little conservation; therefore the patient's remarkably low BMI as consequence of this amino acid substitution seems rather speculative. [4] The exchange of leucine to valine at position 385 is conservative. The non reactive aliphatic side chains of leucine and valine that are important for hydrophobic bonds within the protein are not affected. Functional studies of these variants in DGAT2 have to be performed to clarify the effect of the detected variants on body weight regulation.

**Table 2:** Phenotypic characteristics (gender, age, BMI, BMI-SDS) of heterozygous carriers of infrequent variants detected in the genomic region of *DGAT2*

**Mutation**	**Gender**	**Age [years]**	**BMI [kg/m^2^]**	BMI-SDS*
p.V82A	male	12	29.30	3.6
p.R297Q	female	26	16.9	-1.3
	female	23	17.0	-1.6
p.G318S	male	25	19.6	-1.2
	male	20	17.9	-1.9
p.L385V	male	23	20.4	-0.9
c.812A > G (T194T)	female	12	31.4	4.6
c.920T > C (S230S)	male	13	31.5	3.7
-584C > G	female	34	18.4	-1.3
-30C > T	male	24	19.6	-1.4
IVS2–3C > G	male	27	19.5	-1.5
IVS7+23C > T	female	14	35.9	5.4
	female	16	33.8	5.2
IVS7+73C > T	male	12	29.3	3.3
*22C > T	male	21	33.8	4.7

All individuals are heterozygous carries of these variants. * Estimates based on Hebebrand et al., 1996 (53)

There is no indication that the rare synonymous variants might have an effect on body weight regulation. Variant c.-584C > G in the putative promoter region is located in a potential binding site for the transcription factor ARP-1 (COUP-TF II), which might participate in regulation of lipid metabolism and cholesterol synthesis [27] and is assumed to negatively influence PPARα gene transcription [28]. Two variants were detected in untranslated regions (-30C > T in the 5'UTR and *22C > T in the 3'UTR). These variants may influence mRNA stability, but as they are rare, we assumed that they have no major effect on common obesity under a "common disease common variant"-perspective given that the estimated MAF of each variant was 1/186 = 0.54% (95% confidence interval 0.014%...2.96%). The intronic variants IVS2–3C > G, IVS7+23C > T and IVS7+73C > T are also rare and neither affect any consensus splice site nor do they introduce cryptic splice sites. None of the case control and family based association studies showed an association of investigated variants or haplotypes in the genomic region of *DGAT2*.

Starting off with a mutation screen of the coding sequence and the 5'flanking region we were investigating both case control samples and independent samples with families contributing to a linkage peak. However, due to insufficient statistical power to explore the less common variants (MAF < 0.1), our study design only allows evaluation of common variants.

In conclusion, our results do not support the hypothesis of an important role of common genetic variation in *DGAT2 *for the development of obesity in our sample. One may thus speculate that if there is an influence of genetic variation in *DGAT2 *on body weight regulation, it might either be the less common synonymous or non-coding variants that play an important role.

## Methods

### Study subjects

The ascertainment strategy for the extremely obese and underweight study groups was previously described in detail [29]. Briefly, extremely obese German index patients were ascertained at German hospitals specialized in inpatient treatment of extreme obesity in children and adolescents. All index patients had an age- and gender-specific BMI ≥90^th ^percentile as previously determined in a representative German population sample [30]. The BMIs of the underweight students were below the 15th percentile whereas normal weight controls had BMIs between the 40^th ^and the 60^th ^age- and gender-specific percentile. Mean BMI and age and the respective standard deviations are provided below. Written informed consent was given by all participants and, in the case of minors, their parents. This study was approved by the Ethics Committee of the University of Marburg.

The coding exons of *DGAT2*, the predicted promoter region and an additionall non-coding 5' exon were screened in a 'screening sample' (sample 1) comprising 93 extremely obese children and adolescent cases (48.4 % females, mean BMI 34.4 ± 5.0 kg/m^2^; mean age 14.1 ± 2.0 yrs) and 94 healthy underweight controls (36.2 % females, mean BMI 18.5 ± 1.2 kg/m^2^; mean age 25.5 ± 4.0 yrs). Identified sequence variants were genotyped in sample 2, comprising both the initial groups (sample 1) and additional 87 cases (51.7 % females, mean BMI 36.9 ± 7.0 kg/m^2^; mean age 14.6 ± 2.8 yrs) as well as 93 healthy underweight controls (52.7 % females, mean BMI 18.3 ± 1.0 kg/m^2^; mean age 25.7 ± 3.8 yrs). Finally, in order to increase the power to detect association for one variant (-140C > T), sample 2 was further extended (sample 3). Sample 3 comprised a total of 361 extremely obese cases (53.2 % females, mean BMI 34.7 ± 6.3 kg/m^2^; mean age 14.4 ± 2.6 yrs) and a total of 445 control subjects comprising 278 underweight students (50.7 % females, mean BMI 18.2 ± 1.1 kg/m^2^; mean age 25.0 ± 3.7 yrs) and 167 normal weight controls (60.5 % females, mean BMI 21.8 ± 1.1 kg/m^2^; mean age 24.6 ± 2.4 yrs).

To investigate the potential genetic effects of variants in *DGAT2 *on body weight regulation; SNPs rs1017713, rs3060, -9447A > G and -140C > T were genotyped in a family based association analysis, the respective markers were also genotyped in the 165 genome scan families (sample 4) described previously [8] to test for linkage. Sample 4 is independent of samples 1–3. The aim of our study was the investigation of associations of *common DGAT2 *variants with extreme early-onset obesity.

### Promoter prediction and evaluation of gene structure

Promoter sequence was predicted by PromoterInspector, Mammalian Promoter Prediction Software from Genomatix, [31]. Analyses were based on human genome assemblies hg15 and hg16 [32] and the corresponding ENSEMBL genome browser [33]. cDNA clone sequences of Unigene cluster Hs.334305 representative for the human *DGAT2 *gene were downloaded from NCBI [34] and assembled using GAP4 [35]. *DGAT2 *transcripts were aligned to human genomic sequence using Sim4 [36]. Two known human mRNAs mapped to the *DGAT2 *locus in genome assemblies hg15 and hg16. One of these, AB048286 (2,439 bp) formed the basis for RefSeq entry NM_032564, the annotation status of which was provisional. The second mRNA AL834287 (2,347 bp) was 92 bp shorter at its 5'end than AB048286. Nonetheless, both transcripts harbour 8 exons; and as defined by AB048286, the human *DGAT2 *at chr. 11q13.5 covers 32,766 bp with a coding region (CDS) of 1167 bp extending from exon1 to exon8. In the corresponding Ensembl genome browser [33] there were also two transcripts assigned to the *DGAT2 *locus (ENST00000289503, 1,545 bp; ENST00000228027, 2,238 bp). The former entry harboured 8 exons as found in AB048286 while the latter contained only 7 exons, i.e. exon5 was missing which indicated the presence of at least one alternatively spliced *DGAT2 *transcript.

### Sequencing

Human cDNA clone BF979958 was obtained from RZPD [37] and cultured by standard methods [38]. Sequencing was performed using vector primers and BigDye Terminator Cycle Sequencing v2.0 kit (Applied Biosystems, Weiterstadt, Germany). Sequencing reactions were electrophoresed on ABI 3700 automated sequencers. Base calling was performed using phred [39,40]. Sequence assembly was done using phrap [41]. Trace files were inspected visually in GAP4. RT-PCR: Primers located in exons 1 and 8 of *DGAT2 *as defined by reference sequence NM_032564 were used in a nested PCR approach (PCR I: 1F [ACCCTCATAGCCGCCTACTC], 1R [AGGTTAGCTGAGCCACCCAG]; PCR II: 2F [CTCATAGCCGCCTACTCC], 2R [CTAGAACAGGGCAAGCTGGA]) on human multiple tissue cDNA (Clontech, Heidelberg, Germany) or adipocyte mRNA [42]. Omniscript RT Kit (QIAGEN, Hilden, Germany) was used for reverse transcription. PCR products were cloned into pCR2.1-TOPO (Invitrogen, Karlsruhe, Germany). Sequencing of recombinant clones, sequence assembly, trace file inspection and alignment to genomic sequence was done as described above.

### Mutation screen

A mutation screen was performed in the 8 coding exons of human *DGAT2 *and also in the predicted promoter region and a non-coding 5' exon. For PCR amplification, primers corresponding to intron sequences were used in order to detect potential splice site variants [for PCR primers see additional file 2]. Mutation screens of exon 6 and 8 were performed using denaturing high performance liquid chromatography (dHPLC) analysis on a Transgenomic WAVE^® ^system [Transgenomic, Cheshire, UK; 43]. The optimal melting temperatures for separation of homo- and heteroduplices were deduced from the melting temperature of the PCR-amplicon using WAVEmaker software, version 4.0 (Transgenomic, Cheshire, UK). All chromatograms were compared with chromatograms of sequenced wild-type samples. PCR amplicons showing a peak appearance different to the wild-type pattern were sequenced (SeqLab, Göttingen, Germany). To detect mutations in exons 1–5, 7, the promoter region and the non-coding 5' exon standard nonisotopic single-strand conformation polymorphism analyses (SSCP) was performed [44]. 15% acrylamide gels (Q-BIOgene, Heidelberg, Germany; 37.5:1) were run at 600 V for 16 h at 4°C and for 5.5 h at ambient temperature; all gels were silver stained. The sensitivity of dHPLC has been described to be approximately 95% [45] and that of SSCP about 97% when using two temperatures [46]. All SSCP patterns were compared with patterns of sequenced wild-type samples. Samples that showed a pattern different from that of the wild-types were re-sequenced (Seq Lab, Göttingen, Germany). The nomenclature of the described variants follows den Dunnen and Antonarakis [47] and NM_032564.

### Genotyping

High-throughput genotyping for two additional intronic SNPs (rs1017713, rs3060,) as well as for variants -9447A > G and -140C > T entering the family based association studies was performed as described earlier [48] using matrix-assisted laser desorption/ionization time-of-flight mass spectrometry (MALDI-TOF MS). For case control association studies, genotyping of SNPs -9447A > G and c.920T > C was perfomed via tetra-ARMS-PCR [49] [see additional file 3]. For all other SNPs [see additional file 3], PCR with subsequent diagnostic restriction fragment length polymorphism analyses (RFLP) was used. PCR products were run on ethidium bromide-stained 2.5% agarose gels. Positive controls for the variant alleles and a negative control (water) were run on each gel. To validate the genotypes, allele determinations were rated independently by at least two experienced individuals. Discrepancies were resolved unambiguously either by reaching consensus or by retyping. Missings were retyped twice. Genotyping success rate was above 99%. Genotyping of rs3841596, a biallelic trinucleotide repeat was carried out using fluorescence-based semi-automated technique on an automated DNA sequencing machine (LiCor 4200-2; MWG-Biotech, Ebersberg, FRG). Analyses and assignment of the marker alleles were done with ONE-Dscan Version 1.3 software (MWG-Biotech).

### In silico evaluation of non-synonymous variants

To gain information about putative functional relevance of an amino acid substitution, public sequence database [34] was mined for full length mammalian and more distant related DGAT2 orthologs where particular attention was given to species surpassing oil production. These data were utilized to determine the evolutionary conservation of the DGAT2 amino acid sequence. Protein sequence alignment was carried out via Omiga (Oxford Molecular Ltd.). Transmembrane domains were predicted *in silico *[50].

### Statistics

Associations in the case control sample were analyzed by Cochran-Armitage trend test for genotype frequencies and Fisher's exact test for alleles. Family based association analyses were performed using the pedigree transmission disequilibrium test [PDT; 51]. Analyses of linkage disequilibrium (LD) between the investigated polymorphisms as well as haplotype associations in the families were investigated by FAMHAP v16 [e.g. 52]. All reported p-values are nominal. Due to lack of p-values < 0.05 (see below), adjustment for multiple testing was considered unnecessary.

## Abbreviations

BMI: body mass index

CNS: central nervous system

CNTF: ciliary neurotrophic factor

DGAT: diacylglycerol O-acyltransferase homolog

EST: expressed sequence tag

GAD2: glutamate decarboxylase 2

GAL: galanin

MAF: minor allele frequency

MLB LOD: maximum likelihood binomial logarithm of the odd

QTL: quantitative trait locus

SLC6A14: solute carrier family 6 member 14

SNP: single nucleotide polymorphism

TM: transmembrane domain

UCP: uncoupling protein

UTR: untranslated region

## Authors' contributions

SF designed and carried out PCR and mutation screening, performed genotyping and drafted the manuscript. KR and MP performedpromoter prediction, evaluation of gene structure and resequencing. AS performed statistical analyses. HBr and HBi performed in silico evaluation of non-synonymous variants. AKW and RF participated in the study design and helped drafting the manuscript. KK and TM performed the high-throughput genotyping. MW contributed mRNA for sequencing analyses and participated in the study design. AH and JH conceived the study, and participated in its design and coordination and drafted the manuscript. All authors read and approved the final manuscript.

## Supplementary Material

Additional File 1 mRNAs transcribed from human *DGAT2 *locus. list of mRNAs transcribed from human DGAT2 locus, including status, evidence and gene structure defined by mRNA Click here for file

Additional File 2 PCR primer for mutation screen in *DGAT2*. list of PCR primer for mutation screen in DGAT2 exons Click here for file

Additional File 3 Genotyping information: Genotypes were generated via RFLP, tetra-arms PCR [3] and MALDI-TOF. Genotyping information for investigated SNPs including primer, genotyping methods and restriction enzymes Click here for file
