# Supplementary material for: Mutation screen and association studies in the Diacylglycerol O-acyltransferase homolog 2 gene (DGAT2), a positional candidate gene for early onset obesity on chromosome 11q13
Source: BMC Genet. 2007 May 3;8:17. doi: 10.1186/1471-2156-8-17 (PMC1871603; doi:10.1186/1471-2156-8-17)
Supplement: Additional File 1 — mRNAs transcribed from human DGAT2 locus. list of mRNAs transcribed from human DGAT2 locus, including status, evidence and gene structure defined by mRNA [file 1471-2156-8-17-S1.doc]

**Additional file 1:** mRNAs transcribed from human *DGAT2* locus

| **Transcript** | **Status** | **Evidence** | **Gene structure defined by mRNA^*^** |
| --- | --- | --- | --- |
| AB048286 | known, Reference sequence | experimental,  GenBank entry | 8 exons |
| ENST00000228027 | known | experimental,  GenBank entry | 7 exons  alternatively spliced out exon 5 |
| cDNA clone IMAGE 4373955 | novel | *in silico* | 8 exons  alternative exon 1 |
| EST BF979677 ^a^ | novel | EST sequencing,  RT-PCR | exon number not known  alternative internal exon in between exon 1 and 2 |
| RT-PCR product ^b^ | novel | experimental,  adipocyte mRNA | 7 exons  alternatively spliced out exon 2 |

*^*^* Eight exons as defined by AB048286 were used as a reference for the gene structure of *DGAT2.*

^a^ Sequence data was submitted to GenBank (AY780646)

^b^ Sequence data was submitted to GenBank (AY780647)
