# Supplementary material for: Mutation screen and association studies in the Diacylglycerol O-acyltransferase homolog 2 gene (DGAT2), a positional candidate gene for early onset obesity on chromosome 11q13
Source: BMC Genet. 2007 May 3;8:17. doi: 10.1186/1471-2156-8-17 (PMC1871603; doi:10.1186/1471-2156-8-17)
Supplement: Additional File 2 — PCR primer for mutation screen in DGAT2. list of PCR primer for mutation screen in DGAT2 exons [file 1471-2156-8-17-S2.doc]

**Additional file 2:** PCR primer for mutation screen in *DGAT2*

| **Exon** | **Primer** |
| --- | --- |
| ex01 | ex01-F 5´ TGGTTGGGTTCTGTGTTCTG 3´ ex01-R2 5´ TTGTGCTCATGGACACATACAA 3´ |
| Exon 1 | Ex1a-F 5´ GACCAAAAGCGGGACCTT 3´ Ex1a-R 5´ CGGCTAGGACACCTGGAG 3´ |
|  | Ex1b-F 5´ GCCTCTGCTGGGGTCTAGG 3´ Ex1b-R 5´ CTTCTCGCAGGTCCATAACC 3´ |
| Exon 2 | Ex2-F 5´ GACCCCATGACTGGAGAGAA 3´ Ex2-R 5´ ACCTCTTTTGGGGAAGTGGA 3´ |
| Exon 3 | Ex3-F 5´ TGAAGCCCAGTAGGACCTGA 3´ Ex3-R 5´ GCATCCCTAGAATGAGAGGTG 3´ |
| Exon4 | Ex4-F 5´ CACCCCCACCAACTCTGTAT 3´ Ex4-R2 5´ AGC ACA GCC AGT GAC ACA GT 3´ |
| Exon 5 | Ex5-F 5´ CCAGTTTCCTCTGACCCAAG 3´ Ex5-R 5´ AACAGTGCCCAGCAGGAG 3´ |
| Exon 6 | Ex6-F 5´ CCTGACTGTTGCGTCCTTC 3´ Ex6-R 5´ CACTGGAGGGGTGTGTGTGT 3´ |
| Exon 7 | Ex7-F 5´ GAAACTGAAGCCAGTAAGTAGGG 3´ Ex7-R 5´ CATCCCATAGGCTCAATTCC 3´ |
