# Supplementary material for: Mutation screen and association studies in the Diacylglycerol O-acyltransferase homolog 2 gene (DGAT2), a positional candidate gene for early onset obesity on chromosome 11q13
Source: BMC Genet. 2007 May 3;8:17. doi: 10.1186/1471-2156-8-17 (PMC1871603; doi:10.1186/1471-2156-8-17)
Supplement: Additional File 3 — Genotyping information: Genotypes were generated via RFLP, tetra-arms PCR [3] and MALDI-TOF. Genotyping information for investigated SNPs including primer, genotyping methods and restriction enzymes [file 1471-2156-8-17-S3.doc]

**Additional file** 3: Genotyping information: Genotypes were generated via RFLP, tetra-arms PCR (3) and MALDI-TOF

| **Variant** | **Exon** | **Primer** | **Method** | **Enzyme** |
| --- | --- | --- | --- | --- |
| -9447 A>G | ex01 | ex01-iF 5´ TCTGGAGACAGGGGACCAGTGAAGATGA 3´ ex01-iR 5´ TGCCACCTTGCATAGACAGCTGTACGAC 3´ ex01-oF 5´ CAAGTTAAGGAGCTGGGGCCTCATCCTA 3´ ex01-oR 5´ TCACACTGATACCTCTATGCCCAACAACCA 3´ | tetra-arms PCR | * |
| -584C>G | Promoter | Ex1a-F 5´ GACCAAAAGCGGGACCTT 3´ Ex1a-R 5´ CGGCTAGGACACCTGGAG 3´ | RFLP | BspLI |
| -140C>T | 5´UTR | Ex1b-F 5´ GCCTCTGCTGGGGTCTAGG 3´ Ex1b-R 5´ CTTCTCGCAGGTCCATAACC 3´ | RFLP | BstNI |
| -30C>T | 5´UTR | Ex1b-F 5´ GCCTCTGCTGGGGTCTAGG 3´ Ex1b-R 5´ CTTCTCGCAGGTCCATAACC 3´ | RFLP | SacII |
| Val82Ala | Exon 2 | Ex2-F 5´ GACCCCATGACTGGAGAGAA 3´ Ex2-R 5´ ACCTCTTTTGGGGAAGTGGA 3´ | RFLP | RsaI |
| IVS2-3C>G | Intron 2 | Ex3-F 5´ TGAAGCCCAGTAGGACCTGA 3´ Ex3-R 5´ GCATCCCTAGAATGAGAGGTG 3´ | RFLP | BstNI |
| Thr194Thr | Exon 5 | Ex5-F 5´ CCAGTTTCCTCTGACCCAAG 3´ Ex5-R 5´ AACAGTGCCCAGCAGGAG 3´ | RFLP | BsrI |
| S230S | Exon 6 | S230S-iR 5´ CGACCACGATGATGATAGCATTGACA 3´ S230S-oR 5´ ACTGGAGGGGTGTGTGTGTGTGTAGG 3´ S230S-iF 5´ AGACTATTTGCTTTCAAAGAATGGGCGC 3´ S230S-oF 5´ GCTTACACAGAACCAGACAACTCCAAAGG 3´ | tetra-arms PCR | * |
| Gly318Ser | Exon 7 | Ex7-F 5´ GAAACTGAAGCCAGTAAGTAGGG 3´ Ex7-R 5´ CATCCCATAGGCTCAATTCC 3´ | RFLP | Eco147I |
| p.R297Q | Exon 7 | Ex7-F 5´ GAAACTGAAGCCAGTAAGTAGGG 3´ Ex7-R 5´ CATCCCATAGGCTCAATTCC 3´ | RFLP | BccI |
| IVS7+73C>T | Intron 7 | Ex7-F 5´ GAAACTGAAGCCAGTAAGTAGGG 3´ Ex7-R 5´ CATCCCATAGGCTCAATTCC 3´ | RFLP | BsmAI |
| IVS7+23C>T | Intron 7 | Ex7-F 5´ GAAACTGAAGCCAGTAAGTAGGG 3´ Ex7-R 5´ CATCCCATAGGCTCAATTCC 3´ | RFLP | StyI |
| rs3841596 | Intron 7 | rs3841596-F 5´ CCACTGTTGGTAAGCCCCTA 3´ -IRD 800 rs3841596-R 5´ TCACAGAGCTTGGTTCATCC 3´ | Licor | * |
| Leu385Val | Exon 8 | Ex8-F 5´ CAGGGGAAGGGTGTTGACTA 3´ Ex8-R 5´ GGCAAGCTGGAAGTGAAAGA 3´ | RFLP | BstNI |
| *22C>T | 3´UTR | Ex8-F 5´ CAGGGGAAGGGTGTTGACTA 3´ Ex8-R 5´ GGCAAGCTGGAAGTGAAAGA 3´ | RFLP | BstNI |
